# Crop and Livestock Diversity Cultivating Gastronomic Potential, Illustrated by Sensory Profiles of Landraces

**DOI:** 10.1111/1750-3841.14582

**Published:** 2019-04-08

**Authors:** Magnus Westling, Matti W. Leino, Asgeir Nilsen, Stefan Wennström, Åsa Öström

**Affiliations:** ^1^ Authors are with the School of Hospitality, Culinary Arts and Meal Science Örebro Univ. Sörälgsvägen 1–2 712 60 Grythyttan Sweden; ^2^ Stockholm Univ. Dept. of Archaeology and Classical Studies 106 91 Stockholm Sweden

**Keywords:** culinary diversity, cultivar, cultivated diversity, gastronomy, variety

## Abstract

Landraces, that is, crop and livestock not improved by formal breeding, are scarce in the industrialized world and are mainly maintained *ex situ* for breeding purposes. The natural biodiversity of these landraces may contribute to securing food production that can adapt to a changing climate, crop pathogens, diseases, and other agricultural challenges. In addition, landraces might also possess unique quality traits. Our aim is to take the idea of crop and livestock diversity further by connecting flavor differences of different landraces and varieties, with gastronomic applications. Do landraces provide a creative possibility of using distinct sensory characteristics to create new dishes and food products and/or to optimize recipes by finding the right variety for existing dishes and food products? This study suggests that apple, pea, pear, and poultry landraces, apart from being valuable in terms of biodiversity in sustainable food systems, also possess unique and distinct gastronomic potential. For example, citrus odors in apples, nutty taste in gray peas, astringent taste in pears, and high odor intensity of stable in poultry is of culinary relevance when working with apple juice, plant‐based alternatives to meat, poached pears, and roasted rooster, respectively. To fully explore, and take advantage of, the gastronomic potential landraces possess, additional studies are needed in order to find suitable cooking methods and development of recipes.

**Practical Application:**

Seeking to increase market interest for landraces, highlighting gastronomic values could stimulate higher demand and, in turn, contribute to larger and more resilient populations preserved *in situ*. Specifically, the paper is of use to (I) crop and livestock producers and food companies who wish to provide products with greater sensory variation, (II) individuals, companies, and organizations with the aim to increase landrace demand and/or preservation, and (III) breeders and genetic engineers managing genetic traits of landraces and other varieties.

## Introduction

Since the early 1960s, the overall food system has continually increased its total amount of energy produced, but it has also contributed to pushing biodiversity, along with other planetary boundaries, out of the safe operating space of the biosphere (Gordon et al., 2017). Looking forward, Pimm and Raven (2017) estimate that “half of all species, most of them unknown at the time of their loss, may disappear within the remainder of this century [21st century].” The loss of biodiversity is real, vast, continuing, and irreversible and the main driver of the loss of biodiversity is food systems (Butchart et al., 2010; Gordon et al., 2017; Pimm & Raven, 2017; Steffen, Broadgate, Deutsch, Gaffney, & Ludwig, 2015).

The Food and Agriculture Organization of the United Nations states that “only 30 crops now provide 95 percent of human food‐energy needs and just five of them—rice, wheat, maize, millet and sorghum—provide about 60 percent” (Commission on Genetic Resources for Food and Agriculture, 2017). The energy intake of humans in the form of food is therefore based on relatively few crops. In addition, the diversity *within* crop species today is becoming all more uniform (Van de Wouw, Kik, van Hintum, van Treuren, & Visser, 2009). In contrast, landraces, defined as crops that have “historical origin, distinct identity and lack formal crop improvement, as well as often being genetically diverse, locally adapted and associated with traditional farming systems” (Villa, Maxted, Scholten, & Ford‐Lloyd, 2005) are much more genetically diverse. However, seed from landraces are often found in limited quantities and may vary with regard to harvest quality and, likewise, the genetic population of a given landrace breed may be small and maintaining the landrace without inbreeding depression is often challenging. In Sweden, landraces are mainly preserved *ex situ* as a genetic resource for breeding (see, for example, NordGen, 2017; The Swedish Univ. of Agricultural Sciences, 2016) and only rarely cultivated “on farm.” These landraces may contribute to securing food production that can adapt to a changing climate (Scherr & McNeely, 2008), with warmer winters, changes in precipitation patterns, and new pests in Sweden (Swedish Commission on Climate and Vulnerability, 2007). Likewise, landrace animals carry properties which could be useful in current and future climate change (Hoffmann, 2010). Nonetheless, landraces, particularly with regard to poultry, have been replaced by industrial lines used in highly standardized production conditions, resulting in an erosion of genetic resources (Groeneveld et al., 2010) and a standardization of products (Notter, 1999).

In the project “Gastronomic regions,” carried out by the Swedish Board of Agriculture on behalf of the Swedish Government, gastronomic properties were described as the origin of the foodstuffs in relation to their flavor and cultural conditions (Smaka Sverige, 2015). This description is similar to the description of gastronomic potential and gastronomic properties given by Mithril et al. (2012) emphasizing palatability, cultural heritage, and terroir. Klosse (2014) uses the concept of culinary possibilities, which we consider to be a central part of *gastronomic potential*, to give the idea that different landraces and varieties have different flavors and textures and can therefore be used in different ways, with different cooking methods and recipes.

Gastronomic potential is operationalized in this article as the culinary possibilities of the foodstuffs, based on its distinct sensory characteristics. The aim is to (I) study the sensory variation between different landraces and some varieties of apple, pea, pear, and poultry; and (II) to apply the findings in terms of culinary possibilities. The objective is to support the idea of working toward a cultivated diversity and, thereupon, receive a unique and distinct gastronomic potential. Our hypothesis is that cultivated diversity generates a range of flavors and textures to advance in recipe development. It is our belief that chefs and others evaluating foodstuffs can contribute to an increased crop and livestock diversity, and also a diversity of flavors and textures, by considering culinary possibilities of different landraces and varieties in their kitchen and in other food processing environments.

## Materials and Methods

### Products

This study includes the sensory evaluation of 12 apple, 7 pea, 6 pear, and 6 poultry landraces/varieties (Table 1). Eleven apple, six pea, and five pear landraces/varieties were gathered from small‐scale farms in the mid‐eastern and mid‐western parts of Sweden. Five poultry landraces/varieties were reared at an agricultural school under the same conditions, consuming a combination of complete feeds, pasture, and oats, and slaughtered at the age of 22 weeks at a commercial slaughter house. One apple (“Discovery”), one pea (“Yellow pea”), and one pear (“Conference”) variety, and one broiler (“Ross 308”), were purchased from a local grocery store.

**Table 1:** Apple, pea, pear, and poultry landraces and varieties included in the study

Species	Name	Cultivar/breed type^a^
*Malus domestica* Borkh.	“Antonovka”	Landrace
*Malus domestica* Borkh.	“Bergianäpple”	Landrace
*Malus domestica* Borkh.	“Discovery”	Variety
*Malus domestica* Borkh.	“Katja”	Variety
*Malus domestica* Borkh.	“Mio” from Halland	Variety
*Malus domestica* Borkh.	“Mio” from Stockholm	Variety
*Malus domestica* Borkh.	“Munthe's rosenäpple”	Landrace
*Malus domestica* Borkh.	“Oranie”	Landrace
*Malus domestica* Borkh.	“Sparreholm”	Landrace
*Malus domestica* Borkh.	“Suislepp”	Landrace
*Malus domestica* Borkh.	“Södermanlandskalvill”	Landrace
*Malus domestica* Borkh.	“Tersmeden”	Landrace
*Pisum sativum* var. *arvense* (L.) Poir.	“Hälsinge”	Landrace
*Pisum sativum* var. *arvense* (L.) Poir.	“Retrija”	Variety
*Pisum sativum* var. *arvense* (L.) Poir.	“Rättvik”	Landrace
*Pisum sativum* var. *arvense* (L.) Poir.	“Solberga”	Landrace
*Pisum sativum* var. *arvense* (L.) Poir.	“Sparlösa”	Landrace
*Pisum sativum* L.	“Yellow pea”	Variety
*Pisum sativum* L.	“Östgöta gulärt”	Landrace
*Pyrus communis*	“Blodpäron”	Landrace
*Pyrus communis*	“Clara Frijs”	Landrace
*Pyrus communis*	“Conference”	Variety
*Pyrus communis*	“Lybeckerbergamott”	Landrace
*Pyrus communis*	“Norabergamott”	Landrace
*Pyrus communis*	“Williams”	Variety
*Gallus gallus domesticus*	“Araucana” (rooster)	Landrace
*Gallus gallus domesticus*	“Hedemora” (rooster)	Landrace
*Gallus gallus domesticus*	“Leghorn” (hen)	Variety
*Gallus gallus domesticus*	“Leghorn” (rooster)	Variety
*Gallus gallus domesticus*	“Rhode Island Red” (rooster)	Variety
*Gallus gallus domesticus*	“Ross 308” (hen)	Industrial line
*Meleagris gallopavo*	“Bronze Turkey” (rooster)	Variety

^a^Landraces are defined as cultivars not produced by formal plant breeding, that is, planned crossings and systematic selection, in contrast to varieties. For details on pea landraces, see Nygårds and Leino (2013). For details on apples and pears landraces, see Nilsson (1986, 1989). For details on poultry landraces and varieties, see Al‐Nasser (2007), Boonen (2009), and Olsson (2013).

### Preparation of samples

At least 12 apples, 130 peas, 12 pears, and 3 poultry of each landrace/variety were served to the sensory panel. The apples, pears, and poultry were stored in refrigerators at +4 °C, and the peas (dried) were stored in the dark at room temperature. The apples and pears were served raw. Two days before analysis peas were soaked for 12 hr and boiled until they had a dense but soft texture, which was evaluated by tasting frequently during the boiling process. The yellow peas boiled for 30 min, the gray peas “Retrija,” “Solberga,” and “Sparlösa” 45 min, and the gray peas “Hälsinge” and “Rättvik” boiled for 60 min. The peas were then stored in refrigerators at +4 °C. The poultry breasts were removed, skinned, and cooked at 64 °C sous vide using a steam oven for 3 hr, and then stored in refrigerators at +4 °C. The serving sizes for each evaluation were as follows: one apple/pear slice with peel (each apple/pear was cored and cut into 12 equally sized slices); 5 to 10 peas, depending on the size of the landrace/variety; and a 10 to 15 g sliced piece of cooked poultry breast. The assessors were instructed to decide for themselves how much they wanted to taste. The assessors were allowed to, but not forced to spit. Drinking water as a palate cleanser was available to the assessors throughout the test. All samples were served at room temperature (19 to 22 °C) on white paper plates, in a sensory laboratory conforming to ISO 8589:2007, within a week from delivery day, the cooked peas and poultry were served within 24 hours.

### Sensory evaluation

The sensory evaluation using RGM (Hersleth, Berggren, Westad, & Martens, 2005; Varela & Ares, 2014) was conducted with 26 undergraduate students at the School of Hospitality, Culinary Arts and Meal Sciences, Örebro Univ., who participated voluntarily. The students received information of the study and they had to accept participation of the study before any data collection was carried out. The students were treated as a group of consumers that have experience in sensory evaluation with lectures in physiology of the senses, perception, and sensory methods. The method was conducted in three steps: (1) brief introduction to the assessors, (2) vocabulary generation by triads in groups and individually, and (3) intensity assessment of the samples.

The brief introduction to the assessors was carried out in groups with information on how the tests were to be conducted and on the purpose of the study. In order to facilitate the work with the triads in the second step, the sensory panel obtained examples of sensory characteristics categorized by odor, taste, and texture. These examples were generated by the panel leader (corresponding author of this article), together with two sensory scientists (coauthors of this article), by evaluating the samples used in this study. Odor intensity, taste intensity, and sweet (taste) were used as examples of sensory characteristics for all samples. Grassy (odor), sour (taste), and crispy (texture) were used for apples. Earthy (odor), nutty (taste), and mealy (texture) were used for peas. Honey (odor), astringent (taste), and grainy (texture) were used for pears. Herby (odor), iron (taste), and stringy (texture) were used for poultry.

The vocabulary generation was first carried out in groups to normalize and standardize the groups’ vocabulary and then individually to identify the individual student's sensory characteristics for the samples. Three samples, codified with the letters A, B, and C, were presented in a triad (a set of three samples) with the information that two of the samples were more alike in terms of sensory characteristics. The sensory panel was then asked to describe, in groups, with sensory characteristics categorized by odor, taste, and texture, how the two samples resembled each other and how they differed from the third. Then, six to nine samples, codified with the letters A, B, C, D, and so on, were presented in triads (sets of three samples) to individual students with the information that two of the samples were more alike in terms of sensory characteristics. One sample of each triad was carried over to the next triad. The sensory panel was then asked to describe, individually, with sensory characteristics categorized by odor, taste, and texture, how the two samples resembled each other and how they differed from the third.

The intensity assessment of the samples was carried out after a 10‐min break. The sensory panel was asked to evaluate all samples according to their own set of sensory characteristics from the vocabulary generation, using an intensity scale ranging from 1 to 9, from the lowest intensity (value 1.0) to the highest intensity (value 9.0). Replicates, as questioned by Peltier, Mammasse, Visalli, Cordelle, and Schlich (2018), were not used, similar to Hersleth et al. (2005). The samples were served in randomized order, numbered with a three‐digit code (for example, 453).

### Data analysis

Sensory characteristics that occurred fewer than five times were removed from further data analysis (Hersleth et al., 2005). The analysis was performed by EyeOpenR version 4.1.11 (https://eyequestion.nl by Logic8 B.V., Netherlands) using Multiple Factor Analysis (MFA) with unstandardization of the data for individual Principal Component Analysis (PCA). This analysis generates a score plot and a loading plot, whereas the distances in the score plot reflect the similarities between the samples (Abdi, Williams, & Valentin, 2013; EyeOpenR, 2017). To facilitate the interpretation of the importance of the sensory characteristics for the explained variance between the samples and to understand the samples’ positions, sensory characteristics that had correlations equal to or greater than 0.7 (Guerrero, Gou, & Arnau, 1997) with the first two dimensions of the common space generated by MFA were divided into positive and negative zones of respective dimension and summarized in a table, similar to González‐Tomás and Costell (2006).

## Results and Discussion

### Sensory characteristics used to describe the landraces and varieties

The sensory characteristics odor intensity, sweet, and taste intensity were used for all species (Table 2). The sensory characteristics odor intensity, earthy, bitter, sweet, and taste intensity were used for all crops (Table 2). The most frequently used sensory characteristics to describe odor, taste, and texture, respectively, were odor intensity, sweet, and crispy (Table 2). The sensory characteristics sour, crispy, sweet, mealy, and juicy were used by more than half of the assessors to describe apples (Table 2). The sensory characteristics dry, mealy, and sweet were used by more than half of the assessors to describe peas (Table 2). The sensory characteristics sweet, sour, astringent, bitter, juicy, and crispy were used by more than half of the assessors to describe pears (Table 2). The sensory characteristics odor intensity and sweet were used by more than half of the assessors to describe poultry (Table 2).

**Table 2:** The sensory characteristics of apples, peas, pears, and poultry

Species	Odor	Taste	Texture
Apple	Odor intensity (9)	Sour (25)	Crispy (22)
	Earthy (8)	Sweet (21)	Mealy (17)
	Citrus (7)	Bitter (13)	Juicy (16)
	Grassy (7)	Taste intensity (7)	Solid (6)
			Hard (5)
Pea	Odor intensity (9)	Sweet (14)	Dry (16)
	Earthy (6)	Taste intensity (11)	Mealy (16)
		Nutty (7)	Chewing resistance (12)
		Bitter (6)	
		Earthy (6)	
Pear	Odor intensity (11)	Sweet (25)	Juicy (16)
	Citrus (5)	Sour (19)	Crispy (14)
	Honey (5)	Astringent (17)	Mealy (11)
		Bitter (16)	Hard (8)
		Grassy (6)	Grainy (7)
		Taste intensity (5)	Dry (5)
Poultry	Odor intensity (17)	Sweet (17)	Stringy (12)
	Herby/spicy (8)	Taste intensity (13)	Dry (9)
	Stable (5)	Iron/blood (10)	Juicy (9)
		Bouillon (7)	Crumbly (8)
		Sour (6)	Tender (8)
		Salty (5)	Tough (7)
			Chewing resistance (6)
			Soft (5)

The number of times a sensory characteristic was generated is in parentheses.

### Sensory differences

**Figure 1:**
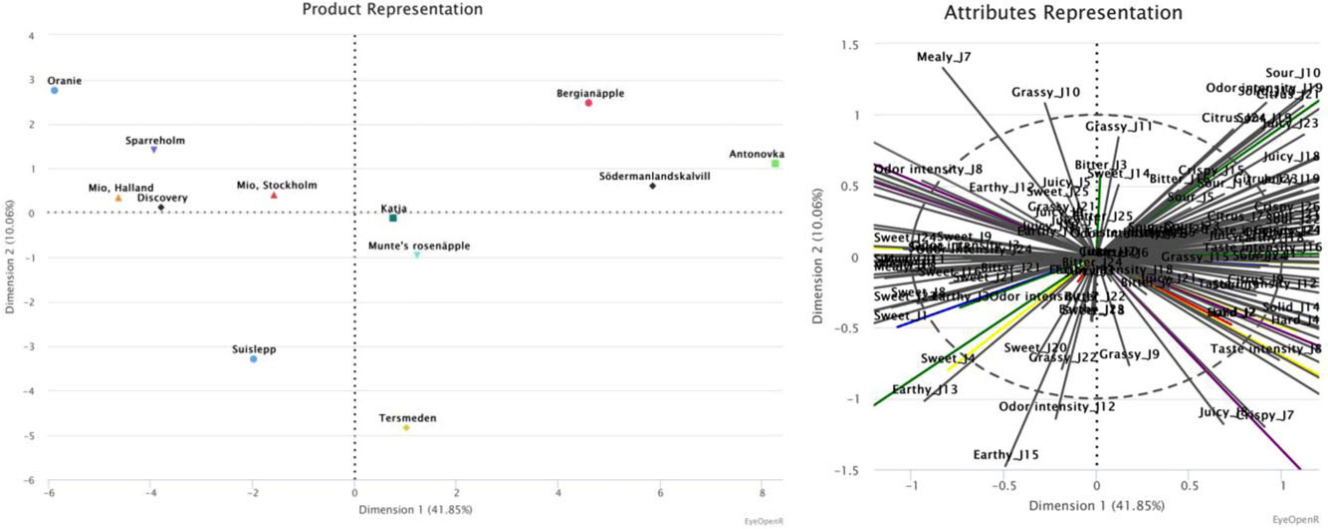
The apples’ positions are defined, by the first two dimensions by MFA. The first dimension explains 41.85% of the variance, the second dimension 10.06%, 51.91% in total.

Based on the results on apples (Table 3; Figure 1), dimension 1 explains 42% of the variance and separated the samples mainly by texture (crispy, mealy, hard, and solid) and taste (sour, sweet, and bitter). This is visualized by the PCA (Figure 1), along the horizontal axis, where the different apples range from being mealy and sweet, to the left in the plot, to the more crispy, sour, hard, solid, and bitter apples to the right of the plot. The mealy and sweet apples also have a high odor intensity, while the crispy, sour, hard, solid, and bitter apples have an overall high taste intensity with citrus and grassy odors. In the second dimension, which explains 10% of the variance, separated the samples by texture (mealy and crispy). This is visualized by the PCA (Figure 1), along the vertical axis, where the different apples range from being mealy, at the top of the plot, to the crispier apples at the bottom of the plot.

**Table 3:** Sensory characteristics of apples, peas, pears, and poultry that have correlations equal to or greater than 0.7 with the first two dimensions of the common space generated by MFA

Species	Dimension	Correlation (+/−)	Odor, taste, and texture, sequentially
Apple	1	+	Crispy (15), sour (13), hard (5), solid (5), bitter (3), taste intensity (2), citrus (1), grassy (1), juicy (1), sweet (1).
		−	Mealy (10), sweet (5), odor intensity (2), juicy (1).
	2	+	Mealy (1).
		−	Crispy (1).
Pea	1	+	Chewing resistance (7), mealy (3), sweet (2), dry (1), earthy odor (1), earthy taste (1).
		−	Mealy (2), odor intensity (2), earthy odor (1), sweet (1).
	2	+	Mealy (4), sweet (3), taste intensity (3), dry (1), nutty (1).
		−	Dry (1), earthy taste (1), odor intensity (1), taste intensity (1).
Pear	1	+	Astringent (9), bitter (6), sour (4), grainy (2), citrus (1), taste intensity (1).
		−	Juicy (4), sweet (3), crispy (2), grassy (2), hard (1), taste intensity (1).
	2	+	Bitter (2), astringent (1), crispy (1), hard (1), mealy (1), sour (1).
		−	Mealy (3), grainy (1), grassy (1), odor intensity (1), sweet (1), taste intensity (1).
Poultry	1	+	Tough (3), chewing resistance (2), stringy (2), sweet (1).
		−	Juicy (3), sweet (2), tender (2), bouillon (1), crumbly (1), dry (1), salty (1), soft (1).
	2	+	Herby/spicy (2), iron/blood (2), dry (1), sweet (1), taste intensity (1), tender (1).
		−	Odor intensity (4), crumbly (1), stable (1), sweet (1), tough (1), taste intensity (1).

The number of times the correlation was generated is in parentheses.

**Figure 2:**
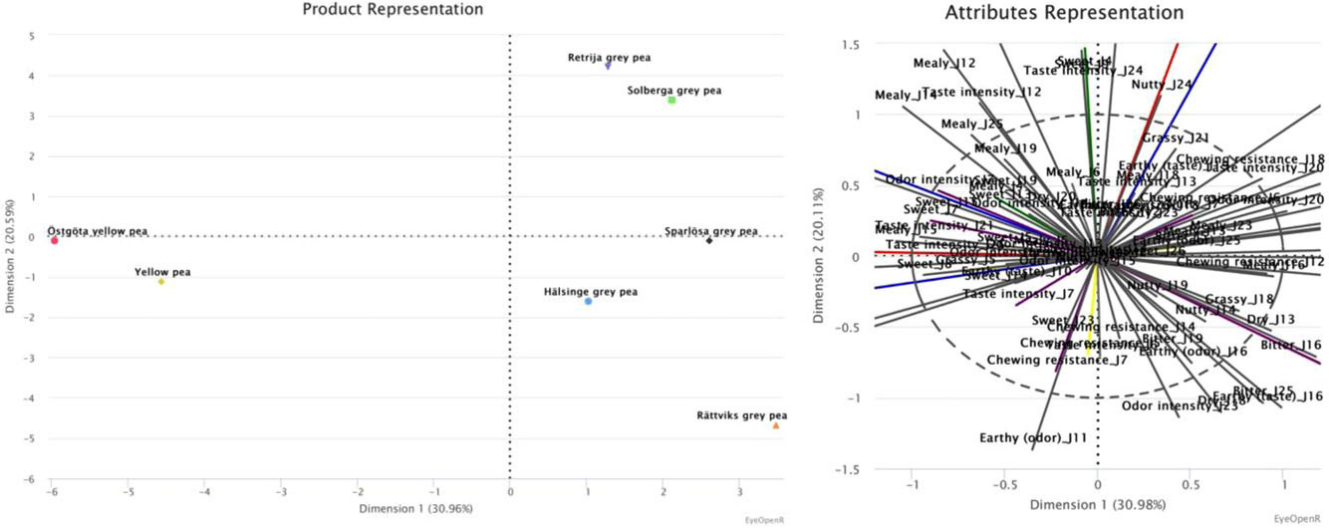
The peas’ positions are defined, by the first two dimensions by MFA. The first dimension explains 30.96% of the variance, the second dimension 20.59%, 51.55% in total.

Based on the results on peas (Table 3; Figure 2), dimension 1 explains 31% of the variance and separated the samples mainly by texture (chewing resistance). This is visualized by the PCA (Figure 2), along the horizontal axis, where the different peas range from having a lower chewing resistance, to the left in the plot, to the peas with a higher chewing resistance to the right of the plot. The peas with a higher chewing resistance also have an earthy taste and a dry texture, while the peas with a lower chewing resistance have a higher odor intensity. In the second dimension, which explains 21% of the variance, separated the samples mainly by texture (mealy) and taste (sweet). This is visualized by the PCA (Figure 2), along the vertical axis, where the different peas range from being mealy and sweet, at the top of the plot, to the less mealy and less sweet peas at the bottom of the plot. The mealy and sweet peas also have a nutty taste, while the less mealy and less sweet peas have an earthy taste and an overall high odor intensity.

**Figure 3:**
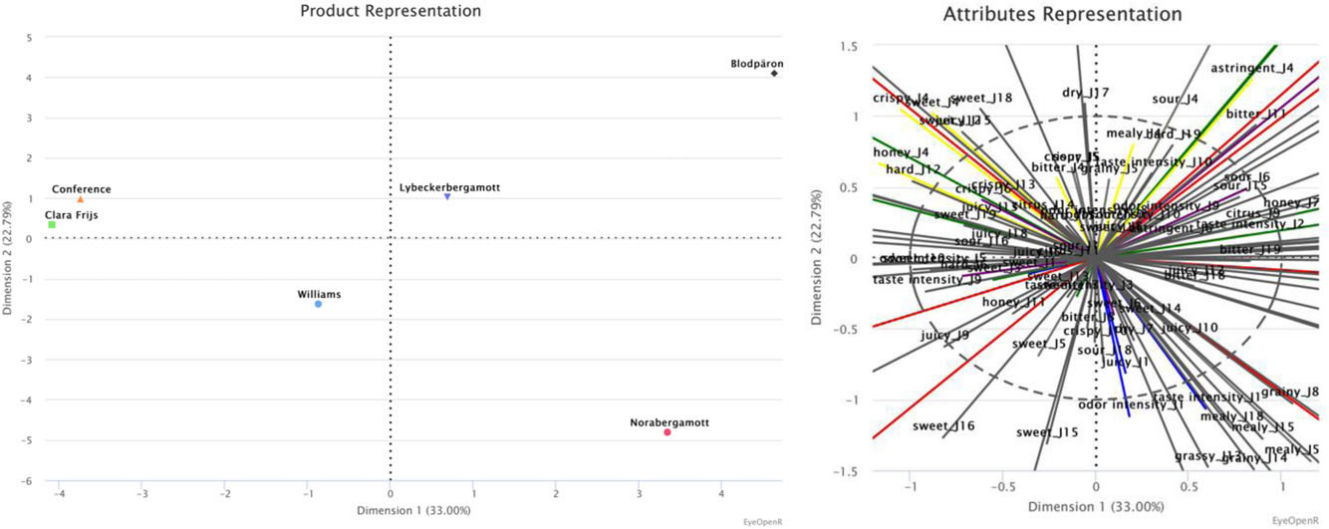
The pears’ positions are defined, by the first two dimensions by MFA. The first dimension explains 33.00% of the variance, the second dimension 22.79%, 55.79% in total.

Based on the results on pears (Table 3; Figure 3), dimension 1 explains 33% of the variance and separated the samples mainly by taste (astringent, bitter, sour, and sweet) and texture (juicy). This is visualized by the PCA (Figure 3), along the horizontal axis, where the different pears range from having an astringent, bitter, and sour taste, to the right in the plot, to the pears with a sweet taste and juicy texture to the left in the plot. The pears with an astringent, bitter, and sour taste also have a grainy texture and citrus odor, while the sweet and juicy pears have a crispy and hard texture and grassy taste. In the second dimension, which explains 23% of the variance, separated the samples mainly by texture (mealy). This is visualized by the PCA (Figure 3), along the vertical axis, where the different pears range from being mealy, at the bottom of the plot, to the less mealy pears at the top of the plot. The mealy pears also have a sweet and grassy taste with an overall high taste and odor intensity, and a grainy texture, while the less mealy pears have a bitter, astringent, and sour taste with a crispy and hard texture.

**Figure 4:**
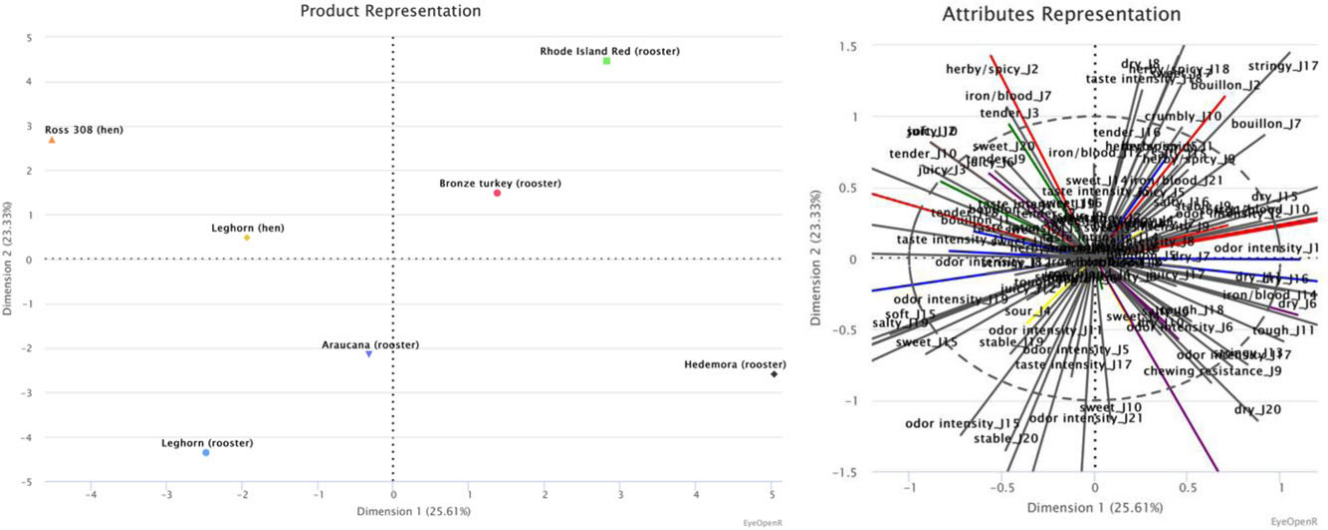
The poultry positions are defined, by the first two dimensions by MFA. The first dimension explains 25.61% of the variance, the second dimension 23.33%, 48.94% in total.

Based on the results on poultry (Table 3; Figure 4), dimension 1 explains 26% of the variance and separated the samples mainly by texture (juicy and tough). This is visualized by the PCA (Figure 4), along the horizontal axis, where the different poultry range from having a juicy texture, to the left in the plot, to the poultry with a tough texture to the right of the plot. The poultry with a juicy texture also have a tender, crumbly, dry, and soft texture and a salty taste of bouillon, while the tough poultry have a stringy texture with high chewing resistance. In the second dimension, which explains 23% of the variance, separated the samples mainly by odor (odor intensity). This is visualized by the PCA (Figure 4), along the vertical axis, where the different poultry range from having a higher odor intensity, at the bottom of the plot, to the poultry with a lower odor intensity at the top of the plot. The poultry with high odor intensity also have stable odors and a tough and crumbly texture, while the poultry with lower odor intensity have iron/blood and herby/spicy taste and a tender and dry texture.

### Gastronomic applications of research findings

The above‐mentioned set of flavors may be needed for chefs and others evaluating foodstuffs in order to optimize recipes and finding the preferred variety for a certain food product. Beyond mere flavors, there is also an interest in recovering landraces among internationally known chefs, as shown in “An Open Letter to the Chefs of Tomorrow” where they suggest that chefs have a responsibility to “help protect the earth's biodiversity, as well as to preserve and create flavors […]” (Bianchi, 2011). In line with these ideas, we suggest the following gastronomic applications of the apple, pea, pear, and poultry landraces/varieties included in this study.

The apple landraces “Antonovka,” “Södermanlandskalvill,” “Bergianäpple,” “Munthe's rosenäpple,” “Tersmeden,” and variety “Katja” with a sour, bitter, and an overall high taste intensity with citrus and grassy odors are desirable when making apple juice. This is based on findings by Jaros, Thamke, Raddatz, and Rohm (2009) who reported a high liking for apple juices with a high acidity, contributing to a refreshing taste. The apple landraces “Oranie,” “Sparreholm,” “Suislepp,” and variety “Mio” with mealy texture and sweet taste likewise is useful when making applesauce. McGee (2004) explains that soft apples produce finer‐grained purees, which is an important characteristic in applesauce. There are various recipes with apples suggesting to use certain varieties and/or apples with particular sensory characteristics, indicating the importance of having a range of apples with different flavors and textures to be able to adapt the recipes according to their standards and also to consumer preferences. For example, based on the *Tolstoys family recipe of apple jam with Antonovka*, our results show it is possible to replace “Antonovka” with “Södermanlandskalvill” or “Bergianäpple” and receive similar flavor in the end‐product.

The gray pea landrace “Solberga” and variety “Retrija” with sweet and nutty taste can be used in order to develop new products which function as plant‐based alternatives to meat. O'Quinn et al. (2016) concluded that nutty/roasted nut as well as sweet taste, among other flavors, for example, browned/grilled and buttery, are preferred characteristic flavors of meat. Browned/grilled and buttery can be elaborated in a plant‐based alternative to meat by adding certain food products and by using cooking techniques such as frying and roasting. Strauta, Muižniece‐Brasava, and Gedrovica (2015) developed extruded snacks based on the gray pea “Bruno” and concluded that the appearance, color, and size of these extruded snacks need improvement to develop more likeable products. This was the only study we found on sensory aspects and product development of gray peas, suggesting more research on this topic is needed. The gray pea landraces “Rättvik” and “Hälsinge” with an earthy taste and an overall high odor intensity can be utilized in lentil recipes, for example, replacing lentils with “Rättvik” and/or “Hälsinge” in savory recipes such as *braised beluga lentils* and *sausages with puy lentils*. FAO (2016) states the following regarding lentils: “Because of their rather delicate, earthy flavor, lentils work well in a variety of dishes and in almost any type of cuisine.” We consider this statement also to hold true for “Rättvik” and “Hälsinge” due to their similar sensory profiles.

The astringent, bitter, and sour pear landrace “Blodpäron” can be used to refine the recipe *poached pears*, which usually is cooked in spiced red wine. The astringency, bitterness, and sourness would in such case come from the produce, and possibly the red wine can be replaced with pears and/or perry. The pear landrace “Clara Frijs” can be used in the same manner as “Conference” due to their similar sensory profiles, for example, as part of a salad or in a wide range of desserts. “Norabergamott,” with similar flavor profile as “Williams,” is preferable as fresh‐cut pear slices. Taiti et al. (2017) compared “Williams” and Nashi pears, whereas “Williams” had higher sweetness, flavor intensity, and odor intensity, and also higher consumer acceptability, indicating these sensory characteristics are important qualities for consumers when eating fresh‐cut pear slices.

The poultry landrace “Hedemora” with high odor intensity of stable and with a tough and stringy texture can be used to develop the recipe *roasted rooster* with a distinct sensory profile. The odor of stable would be suitable to pair with common ingredients in roasted rooster recipes that balances the odor of stable, such as potato, garlic, onion, butter, and lemon. By placing the rooster in brine and roasting it on low heat for a relatively long time will tenderize the meat. The poultry landrace “Araucana” and variety “Leghorn,” also with high odor intensity of stable, but with a juicier and more tender texture, is of culinary relevance when working with fried or grilled rooster. The juicy and tender texture is an important characteristic when using these cooking techniques. Dyubele, Muchenje, Nkukwana, and Chimonyo (2010) reported that roasted poultry meat receives higher overall flavor intensity than boiled poultry meat, indicating that by using cooking techniques such as roasting, frying, and grilling, the overall odor and taste intensity of the meat increases. The herby/spicy varieties “Rhode Island Red” and “Bronze turkey” with taste of iron/blood and with a tough and stringy texture can be used in flavorful one‐pot poultry stews such as *coq au vin*. The distinct flavors of these varieties should match the rich, earthy, and sweet taste of this traditional dish.

### Sensory variation and cultural applications of landraces

The sensory profile of a landrace can vary due to the variability within the landrace (Harlan, 1975; Zeven, 1998) and due to the terroir effect, defined by De Andrés‐de Prado et al. (2007) as “an amalgamation of influences that include climate, landscape (slope, exposure, and the biological and physical environment), soil, and geology.” Also, the sensory profile can vary due to harvest/slaughter time and storage period (Elgar, Watkins, Murray, & Gunson, 1997; Horsted, Henning, & Hermansen, 2007). Furthermore, there is a seasonal effect, for example, Bunning, Kendall, Stone, Stonaker, and Stushnoff (2010) concluded that differences among lettuce cultivars have larger impact than growing season on sensory profiles, Lynch, Koppel, and Reid (2016) concluded that seasonal differences have a larger impact on black walnut cultivars than cultivar on sensory profiles, and Seppä, Tahvonen, and Tuorila (2016) concluded that even though harvest year have effect on the apples sensory profiles, the effect varies due to the variety. In this study, we do not provide an overview of the interacting influence of weather conditions and handling from field to fork impact on the sensory characteristics. Although the sensory profile may vary, there is—more or less—an effect of landrace/variety on the sensory profiles, as shown in our results. After all, the increase of sensory variation due to a diversity of landraces and varieties in a gastronomic context is crucial to the idea of cultivated and culinary diversity; the variability and variation itself is a gastronomic quality, cultivating gastronomic potential. In addition to sensory characteristics, utility, and purposiveness of certain landraces and varieties, there is a broader ethic of relationality, a localized component, triggered by landraces in general, attributed to local food systems in particular, and, foodwise, based partly on other sensorial and aesthetic qualities (VanWinkle, 2017) than the ones we have addressed in this article. However, even though landraces derive from—and are an important part of—local food systems (Singh, 2018), they are not immediately limited to local food systems, suggesting the results from our article are potentially applicable to other sustainable food systems as well.

## Conclusion

By using an intraspecific diversity of landraces and varieties, chefs and others evaluating foodstuffs have the possibility to receive a comprehensive set of flavors. This study suggests that apple, pea, pear, and poultry landraces, apart from being valuable in terms of biodiversity in sustainable food systems, also possess unique and distinct gastronomic potential. In addition to usage of landraces, a collection of varieties is important in terms of flavor, thus enhancing the intraspecific diversity of a given crop and/or livestock.

Foodstuffs with unexpected flavors may prove to be a challenge to create meals from, since consumers used to certain flavors in particular foodstuffs may show lower preference for them due to false expectations. Additional studies are needed in order to find suitable cooking methods and development of recipes that take full advantage of the gastronomic potential that landraces possess, foodstuff that most often are overlooked in terms of gastronomic value.

## Authors’ Contributions

M. Westling and Å. Öström designed the study. M. Westling collected test data and Å. Öström contributed with the performing of the sensory assessments. M. Westling and A. Nilsen analyzed test data. M. W. Leino contributed with material information. All authors interpreted the results. M. Westling drafted the manuscript and M. W. Leino, A. Nilsen, S. Wennström, and Å. Öström contributed with manuscript writing. M. W. Leino, Å. Öström, and S. Wennström revised the work critically. All authors have read and approved the final version of the manuscript.
